# Prevalence and course of depression in older people with aortic stenosis undergoing transcatheter aortic valve implantation – a systematic review and meta-analysis

**DOI:** 10.1186/s12877-025-06402-w

**Published:** 2025-10-06

**Authors:** Verena Maschke, Ute Mons, Valerie Lohner

**Affiliations:** Department of Cardiology, Cardiovascular Epidemiology of Aging, Faculty of Medicine and University Hospital Cologne, University of Cologne, Kerpener Straße 62, Cologne, 50937 Germany

**Keywords:** Depression, Psychocardiology, Aortic valve stenosis, Transcatheter aortic valve replacement, Meta-analysis, Systematic review

## Abstract

**Background:**

Depression is the most prevalent psychiatric disorder in older people with cardiovascular disease and an independent risk factor for cardiac surgery outcomes. Given the rising number of older people with aortic stenosis in need of transcatheter aortic valve implantation (TAVI) and the increasingly recognized role of depression as a prognostic factor, this systematic review and meta-analysis aimed to derive a global estimate of the prevalence of depression and to examine its course from pre- to post-TAVI.

**Methods:**

We included articles reporting prevalence of depression (diagnosed or assessed using validated instruments) or change in depressive symptoms in people undergoing TAVI. We performed three main meta-analyses: (1) prevalence of diagnosed depression, (2) prevalence of assessed depression and (3) standardized mean change (SMC) of depressive symptoms from pre- to post-TAVI.

**Results:**

We included 32 studies in this systematic review, 26 of which were suitable for meta-analysis. The pooled prevalence of diagnosed and assessed depression was 6.0% [95%-Confidence interval: 3.3, 10.6] and 18.5% [13.0, 25.6], respectively. We observed a small, albeit non-significant trend towards a decrease in depressive symptoms from pre- to post-TAVI (SMC: -0.08 [-0.37, 0.22]).

**Conclusions:**

A relevant proportion of people undergoing TAVI experiences depression, and the discrepancy in prevalence estimates of diagnosed and assessed depression indicates a potential under-diagnosis of depression. Disease management strategies should be adapted to include screening for and adequate treatment of depression in this population. Improvement of depressive symptoms after TAVI should not be taken for granted.

**Supplementary Information:**

The online version contains supplementary material available at 10.1186/s12877-025-06402-w.

## Introduction

Aortic valve stenosis is the most common valvular heart disease in Western countries with a prevalence of about 12% in people over 75 years of age [1]. If left untreated, severe aortic valve stenosis leads to heart failure within a few years, with a 5-year mortality of 94% [2]. In 2002, transcatheter aortic valve implantation (TAVI) was introduced as a minimally invasive treatment option for aortic valve stenosis [3], and recent studies have demonstrated superior or at least similar outcomes compared to surgical aortic valve replacement [4, 5]. According to the 2021 guidelines for the management of valvular heart disease of the European Society of Cardiology and the European Association for Cardio-Thoracic Surgery, TAVI is recommended as the first-line treatment for patients aged 75 years or older with symptomatic severe aortic valve stenosis, as well as for younger patients who are at high surgical risk. This decision is based on a comprehensive assessment by a multidisciplinary cardiac team that takes into account factors such as frailty, comorbidities, anatomical suitability and remaining life expectancy, which should be at least one year. In Germany alone, which performs the highest number of TAVI procedures in Europe [6], about 26,000 procedures are performed annually, representing 77% of all aortic valve implantations [7].

Among people with cardiovascular disease, depression is one of the most common psychiatric disorders, with an estimated global prevalence between 18 and 31% [8, 9], and is linked to lower quality of life and poorer cardiovascular outcomes [10]. In the context of cardiac surgery, findings regarding the trajectories of preoperative depression after treatment are conflicting. Cardiac surgery can be seen as a critical event that can either lead to remission of depressive symptoms due to relief from physical symptoms, or to the persistence or even worsening of depressive symptoms, particularly in case of postoperative complications [11]. Most previous studies observed persistent or decreasing depressive symptoms after cardiac surgery [12, 13]. This variability in depression trajectories is also evident in studies on people with aortic valve stenosis undergoing TAVI. Furthermore, the prevalence of depression in people with aortic valve stenosis undergoing TAVI varies substantially between studies [14].

Therefore, to unify the existing prevalence estimates for depression in older people undergoing TAVI, and to examine the change in depressive symptoms from pre- to post-TAVI, we conducted a systematic literature review and meta-analysis.

## Methods

The protocol of this systematic review and meta-analysis was pre-registered at the International prospective register of systematic reviews (PROSPERO) (*CRD42023389245*). The reporting follows the Preferred Reporting Items for Systematic Reviews and Meta-Analysis (PRISMA) statement [15] (See Supplementary Material A1, Additional File 1 for the PRISMA checklist).

### Search strategy and study selection

We performed a literature search up until 15 January 2023 using PubMed (MEDLINE), Web of Science and APA PsycINFO (See Supplementary Material A2, Additional File 1 for the search strings). An update was carried out before the final analysis on 7 September 2023.

Studies had to provide a prevalence of depression, or scale values for depressive symptoms or data to calculate those from to be eligible. We only considered original articles with a quantitative approach published in peer-reviewed journals. Only articles in English, German or Dutch were eligible due to lack of resources for appropriate translation of other languages. Studies were excluded if they involved study populations with severe disease other than aortic valve stenosis (e.g. cancer), or if they assessed depression with non-validated instruments (i.e., if no published validation study could be found). One reviewer initially screened records based on title and abstract for eligibility. Then, two reviewers independently performed the full-text screening. We resolved disagreements through discussion, involving a third reviewer when necessary.

### Data extraction

Two reviewers independently extracted relevant data from the included studies. Disagreements were resolved through discussion, with a third reviewer resolving any remaining disagreements. We extracted data using a standardized form capturing (1) prevalence and confidence interval (CI) of depression, (2) sample size and number of depression cases, (3) mean/median values of depressive symptoms and their dispersion, (4) pre-post-correlations for mean/median values of depressive symptoms at baseline and follow-up, (5) timing of depression assessment in relation to TAVI, (6) criteria/instruments used for depression measurement and their cut-off value, and (7) proportions of sex, age, and ethnicity. Since previous studies found substantial differences in prevalence estimates between diagnosed and assessed depression [14, 16], we divided our analyses into these two categories. We considered depression to be ‘diagnosed’ when a recognized diagnostic system was used or when cases were identified through medical records (including diagnoses, or treatment with antidepressants), and to be ‘assessed’ if a validated instrument was used to assess depressive symptoms. When studies reported several follow-up measurements, we extracted the latest follow-up values for the outcomes of interest. From extracted sample size and number of depression cases, we calculated 95% Wilson CIs for the prevalence estimates.

### Quality assessment and certainty of evidence

To assess the quality of individual studies at the outcome level, we used the 2020 version of the Joanna Briggs Institute (JBI) Critical Appraisal Checklist for Prevalence Studies [17]. When studies provided no sample size calculation, we conducted our own sample size analysis, as recommended by the JBI Critical Appraisal Checklist. For this, we had to set Z statistic (Z), expected prevalence (P) and precision (d) (Z = 1.96, d = 0.05). We chose expected prevalence of depression based on published global depression prevalence estimates in the general older population, which were 5.7% and 28.4% for diagnosed and assessed depression, respectively [18, 19]. To evaluate the certainty of overall evidence we used the Grading of Recommendations, Assessment, Development and Evaluation (GRADE) method [20]. Two reviewers independently conducted the critical appraisal and certainty of evidence rating, and disagreements were resolved through discussion, consulting a third reviewer when necessary.

### Statistical analyses

#### Pooled prevalence of depression

We employed a random-effects (RE) generalized linear mixed model (GLMM) with logit link and maximum likelihood estimator to calculate the pooled prevalence of diagnosed and assessed depression and the corresponding 95%-CI based on a t-distribution. We chose GLMM because we expected heterogeneity across the included studies, and because of its better performance and easier interpretability compared with two-step methods for transforming prevalence values for meta-analysis [21]. Additionally, we fitted a mixed-effects meta-regression model using a dichotomous moderator to test for differences between the pooled prevalences of diagnosed and assessed depression. In cases where studies reported prevalences of both diagnosed and assessed depression, we only included the prevalence of diagnosed depression to meet the requirement of independent comparison groups.

#### Standardized mean change of depressive symptoms

For meta-analysis of standardized mean change (SMC) from pre- to post-TAVI, median score values and interquartile ranges were converted into an estimated mean and standard deviation using the Box-Cox method [22]. Given that studies measured a single group before and after TAVI, following a single-group pre-test-post-test design, the SMC was calculated using the change score standardisation [23]. For the meta-analysis of SMC we employed a RE model with restricted maximum likelihood estimation, and the Knapp and Hartung method [24] for estimating summary effects. While we had originally planned to use the DerSimonian and Laird estimation method in our study protocol, we ultimately chose the restricted maximum likelihood approach because it is generally preferred due to its tendency to yield approximately unbiased heterogeneity estimates [25]. Since we obtained the pre-post-correlation only from Olszewska-Turek 2022 [26], we assumed the same pre-post-correlation for all included studies (*r* = 0.0358).

Generally, if different studies used the same databases, resulting in overlapping samples, we excluded the studies with the smaller sample size from meta-analysis to ensure independence between samples. We quantified heterogeneity using Cohen’s Q statistic, ${\text{I}}^{2}$ statistic and prediction interval. Additionally, we conducted comprehensive sensitivity analyses to evaluate the robustness of our results against alternative model specifications and assumptions (See Supplementary Material A3, Additional File 1). For data preparation and analysis, we used R (version 4.2.1) [27], and the following packages: *metafor* [28], *lme4* [29]*, estmeansd* [30], and *Hmisc* [31]. We considered a *p*-value < 0.05 to be statistically significant.

## Results

**Fig. 1:**
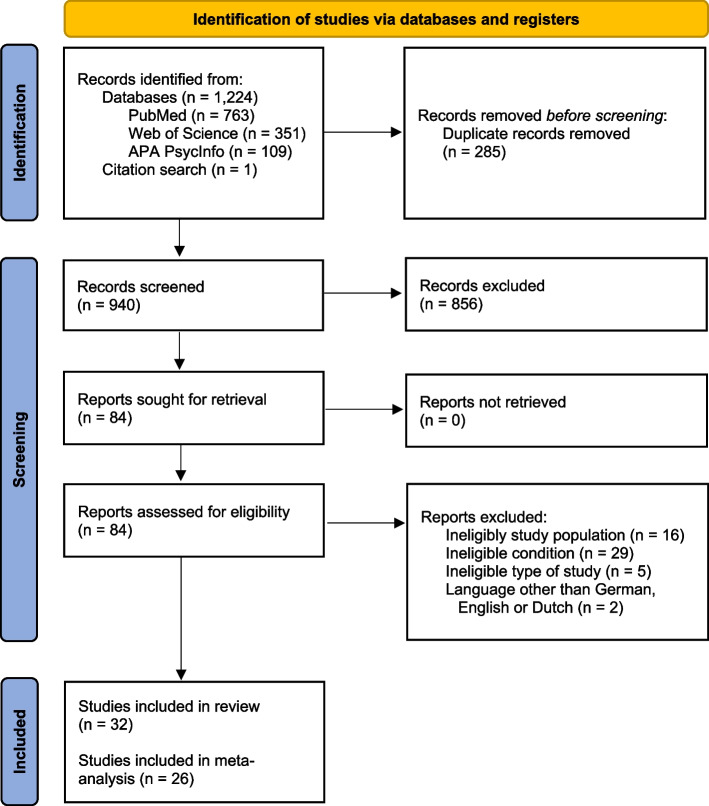
PRISMA 2020 flow diagram [15]

Our search strategy identified 1,225 potentially eligible studies, of which 84 were screened in full text. We reached out to corresponding authors of 17 studies via e-mail, aiming to obtain relevant missing data. Out of these inquiries, we received two responses, of which one provided a pre-post-correlation and one a standard deviation. Finally, we included 32 studies in the review [26, 32–62], of which 26 studies were suitable for meta-analysis [26, 32–37, 40–42, 45–48, 50–53, 56, 57, 60, 62] (see Fig. 1).

### Study characteristics

Table 1 summarizes the characteristics of included studies. These encompassed prospective (*n* = 18) or retrospective (*n* = 12) cohort studies and randomized controlled trials (RCTs) (*n* = 2). Study samples were mostly from European countries (*n* = 16) or the United States of America (USA) (*n* = 9). The mean age of study participants in the included cohorts ranged from 77.8 to 85.1 years, and 20% to 63% were women. Ethnicity of study participants was reported in only five studies, revealing that 86% to 100% of participants were white. Given that all but one of the studies were conducted in Western countries, we assume that the study population is predominantly Caucasian/white.

**Table 1:** Main characteristics of studies included in this systematic review and meta-analysis

Study	Study design	Country	Mean age (SD)	% Women	Sample Size baseline	Sample Size follow-up	Last follow-up in months	Specification of depression	Instrument/definition used	Outcome(s) of interest for meta-analysis
Amonn 2013 [61]	PCS	Switzerland	79.7 (9.2)	49.0	51	31	15	Assessed	HADS ≥ 8	Prevalence follow-up
Annie 2021 [44]	RCS	US	78.1 (9.0)	48.4	15,323	15,323	60	Diagnosed	ICD-10 diagnosis	Prevalence follow-up
Bäz 2021 [43]^a^	PCS	Germany	77.9 (7.5)	54.9	224	-	-	Assessed	HADS ≥ 8	Prevalence baseline
Bäz 2020 [49]^a^	PCS	Germany	77.8 (7.7)	57.1	140	-	-	Assessed	HADS ≥ 8	Prevalence baseline
								Diagnosed	ICD-10 diagnosis	Prevalence baseline
Boureau 2017 [56]	PCS	France	83.7 (4.6)	44.0	150	-	-	Assessed	GDS-4 ≥ 1	Prevalence baseline
								Diagnosed	Treatment with antidepressants	Prevalence baseline
Boureau 2015 [57]	RCS	France	83.7 (4.0)	51.9	131	-	-	Assessed	GDS-4 ≥ 1	Prevalence baseline
Dautzenberg 2021 [42]	PCS	The Netherlands	80.7 (6.2)	52.7	490	-	-	Assessed	GDS-15 ≥ 6	Prevalence baseline
Drudi 2018 [53]^b^	PCS	Canada, US, France	83.7 (5.7)	58.6	657	494	6	Assessed	GDS-5 ≥ 2	Prevalence baseline + follow-up
Edwards 2020 [48]	RCT	US	82.4 (8.5)	43.2	139	-	-	Diagnosed	MINI	Prevalence baseline
			82.3 (7.5)	50.0	64	47	1	Assessed	BDI-II [definition not reported]	SMC
Eide 2023 [62]	PCS	Norway	84.8 (2.8)	63.0	62	53	6	Assessed	HADS ≥ 8	Prevalence baseline + follow-up, SMC
El-Sabawi 2023 [34]	PCS	US	80.8 (8.2)	43.5	873	207	12	Assessed	PHQ-2 ≥ 3	Prevalence baseline + follow-up
Geers 2023 [33]	RCS	Belgium	84.0 (4.0)	61.0	91	-	-	Assessed	GDS-15 ≥ 5	Prevalence baseline
					100	-		Diagnosed	Treatment with antidepressants	Prevalence baseline
Grant 2021 [41]^c^	RCS	US	80.1 (6.7)	47.1	99,400	-	-	Diagnosed	ICD-9 + ICD-10 diagnosis	Prevalence baseline
Imran 2018 [55]	RCS	US	79.2 (1.9)	38.5	23	23	3	Assessed	PHQ-9 diagnosis	Not included
Jafri 2022 [39]	PCS	US	79.0 (10.0)	29.0	21	16	-	Assessed	PHQ-9 diagnosis	Not included
Khan 2019 [51]	PCS	Canada	83.3 (5.7)	39.3	117	-	-	Assessed	PHQ-2 ≥ 3	Prevalence baseline
Kleczynski 2021 [40]	RCS	Poland	80.0 (5.5)	62.0	53	53	12	Assessed	HADS ≥ 8	SMC
			81.0 (4.9)	58.0	52	52	12	Assessed	HADS ≥ 8	SMC
Krittanawong 2020 [47]^c^	RCS	US	80.9 (8.8)	47.5	11,160	-	-	Diagnosed	ICD-9 diagnosis	Prevalence baseline
Lantelme 2020 [46]	RCS	France	82.7 (6.9)	50.1	20,443	-	-	Diagnosed	ICD-10 diagnosis	Prevalence baseline
Newell 2022 [37]	RCS	US	79.8 (7.8)	45.2	87,142	-	-	Diagnosed	ICD-10 diagnosis	Prevalence baseline
Olszewska-Turek 2022 [26]	PCS	Poland	82.1 (6.1)	63.0	131	43	12	Assessed	GDS-15 ≥ 6	Prevalence baseline + follow-up, SMC
Rodighiero 2020 [45]	PCS	Canada	81.4 (6.3)	46.0	233	-	-	Assessed	GDS-5 ≥ 2	Prevalence baseline
Rogers 2018 [54]	RCT	UK	82.0 (4.8)	55.6	27	23	6	Assessed	HADS ≥ 8	Not included
Sathananthan 2019 [50]^b^	PCS	Canada, US, France	83.5 (5.6)	45.0	755	542	12	Assessed	GDS-5 ≥ 2	Prevalence baseline + follow-up
Schofer 2022 [36]	RCS	Germany	82 (78, 85)^d^	55.6	21,430	-	-	Diagnosed	ICD-10 diagnosis	Prevalence baseline
Shah 2019 [52]^c^	RCS	US	81.0 (8.8)	47.3	25,990	-	-	Diagnosed	ICD-9 diagnosis	Prevalence baseline
Sun 2022 [35]	PCS	China	78.6 (4.8)	25.6	78	59	8	Assessed	HADS ≥ 8	Prevalence baseline + follow-up, SMC
Surman 2022 [38]	PCS	Australia	82.9 (6.9)	20.0	100	93	12	Assessed	PHQ-9 [definition not reported]	Not included
Tamm 2023 [32]	PCS	Germany	79.8 (5.6)	40.6	248	118	12	Assessed	PHQ-9 ≥ 10	Prevalence baseline, SMC
Tully 2015 [59]	PCS	Australia	85.1 (4.7)	41.2	42	40	6	Assessed	PHQ-9 ≥ 10	Not included
Völler 2015 [58]	RCS	Germany	80.3 (6.2)	57.9	76	-	-	Assessed	HADS [definition not reported]	Not included
Zanettini 2014 [60]	PCS	Italy	83.5 (5.0)	53.0	58	42	18	Assessed	GDS-30 ≥ 11	Prevalence follow-up

*SD* standard deviation, *PCS* prospective cohort study, *HADS* Hospital Anxiety and Depression Scale, *RCS* retrospective cohort study, *US* United States, *ICD* International Statistical Classification of Diseases and Related Health Problems, *GDS* Geriatric Depression Scale, *MINI* Mini-International Neuropsychiatric Interview, *BDI* Beck Depression Inventory, *SMC* standardized mean change, *PHQ* Patient Health Questionnaire, *RCT* randomized controlled trial, *UK* United Kingdom

^a^Overlapping samples

^b^Overlapping samples

^c^Overlapping samples

^d^Median and interquartile range

### Pooled prevalence of depression among people undergoing TAVI

#### Prevalence of diagnosed depression

**Fig. 2:**
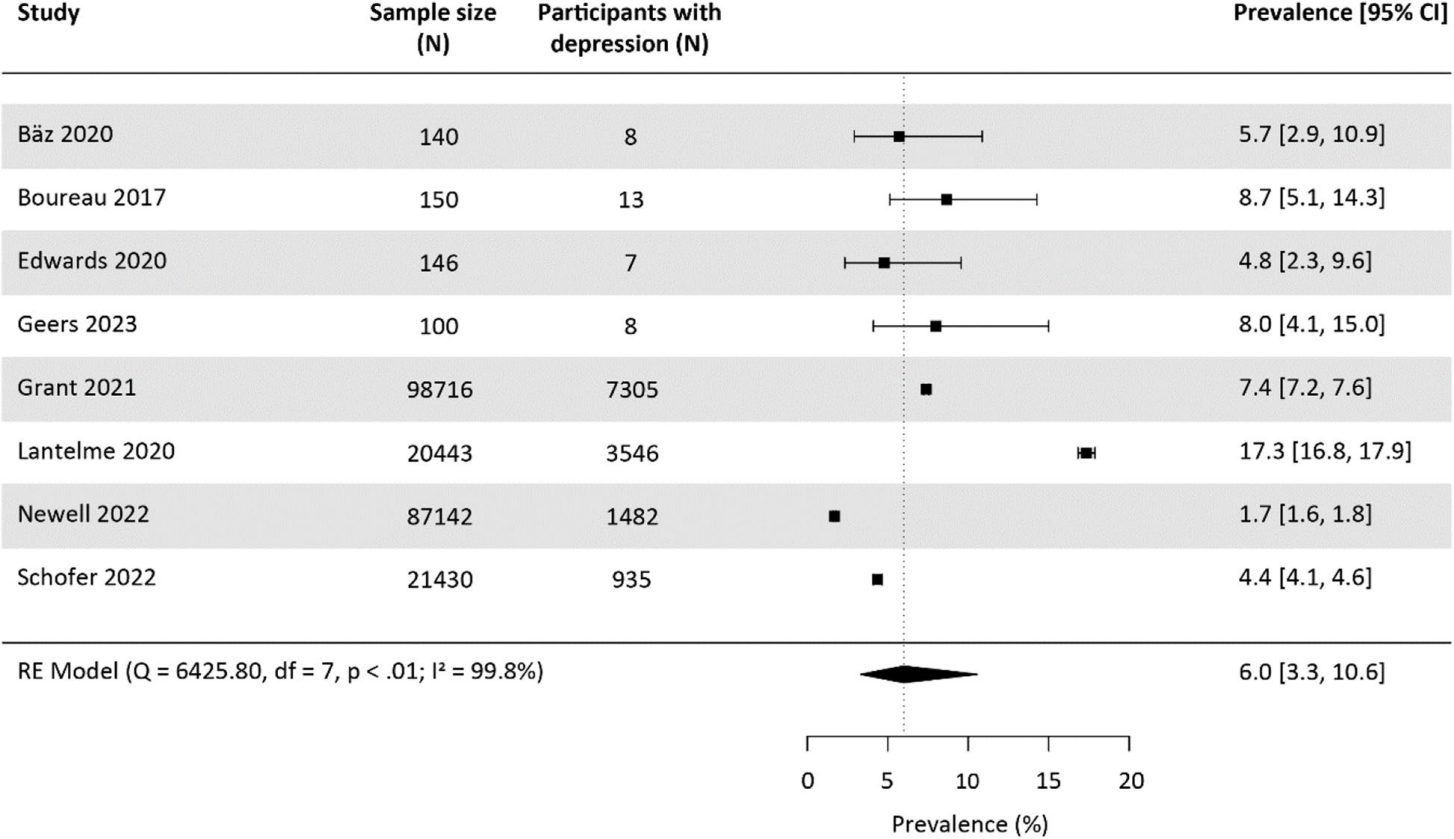
Meta-analysis on the prevalence of diagnosed depression at baseline

Ten studies reported prevalences of diagnosed depression at baseline [33, 36, 37, 41, 46–49, 52, 56], three of which used the same database for analysis, resulting in overlapping samples (Grant 2021 [41], Krittanawong 2020 [47], Shah 2019 [52]). We included only Grant 2021 [41], the most recent study with the largest sample size. Thus, we calculated the pooled prevalence of diagnosed depression at baseline based on two prospective cohort studies, five retrospective cohort studies, and one RCT, encompassing a total of 228,267 study participants. Diagnosed depression was determined based on International Statistical Classification of Diseases and Related Health Problems (ICD) codes (*n* = 4), clinical analysis (*n* = 1), Mini-International Neuropsychiatric Interview (MINI) (*n* = 1) or treatment with antidepressants (*n* = 2). The pooled prevalence of diagnosed depression was 6.0% [95%-CI: 3.3, 10.6] (Fig. 2), with significant heterogeneity between studies and a 95%-prediction interval of 1.1 to 27.0. Among the included studies, only one provided a follow-up prevalence of diagnosed depression. This study, based on ICD codes, reported a prevalence of 13.4% [95%-CI: 12.8, 13.9] in a sample size of 15,323 participants, with a follow-up duration of up to 60 months [44].

#### Prevalence of assessed depression

**Fig. 3:**
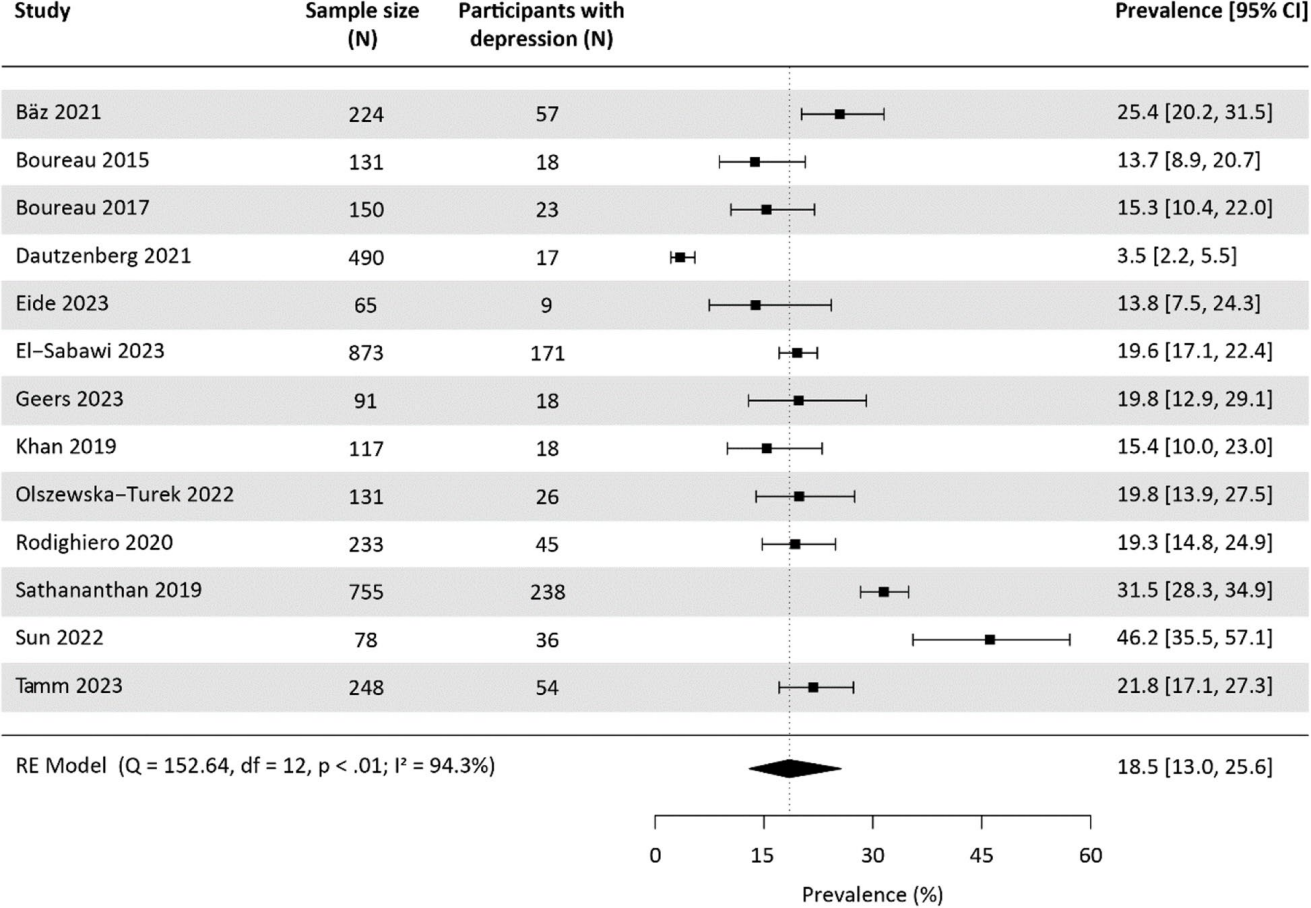
Meta-analysis on the prevalence of assessed depression at baseline

Fifteen studies reported prevalences of assessed depression at baseline [26, 32–35, 42, 43, 45, 49–51, 53, 56, 57, 62]. However, Bäz 2021 [43] and Bäz 2020 [49], and Sathananthan 2019 [50] and Drudi 2018 [53] used the same database, resulting in overlapping samples. We included the most recent studies with the largest sample sizes, Bäz 2021 [43] and Sathananthan 2019 [50]. Thus, our meta-analysis on prevalence of assessed depression before TAVI included 12 prospective and one retrospective cohort studies, encompassing a total of 3,586 study participants. The instruments used for assessing depression were the Hospital Anxiety and Depression Scale (HADS) (*n* = 3), a variant of the Patient Health Questionnaire (PHQ) (*n* = 3) and a variant of the Geriatric Depression Scale (GDS) (*n* = 7). The pooled baseline prevalence of assessed depression was 18.5% [95%-CI: 13.0, 25.6] (Fig. 3), with significant heterogeneity between studies and a 95%-prediction interval of 4.8 to 50.2.

**Fig. 4:**
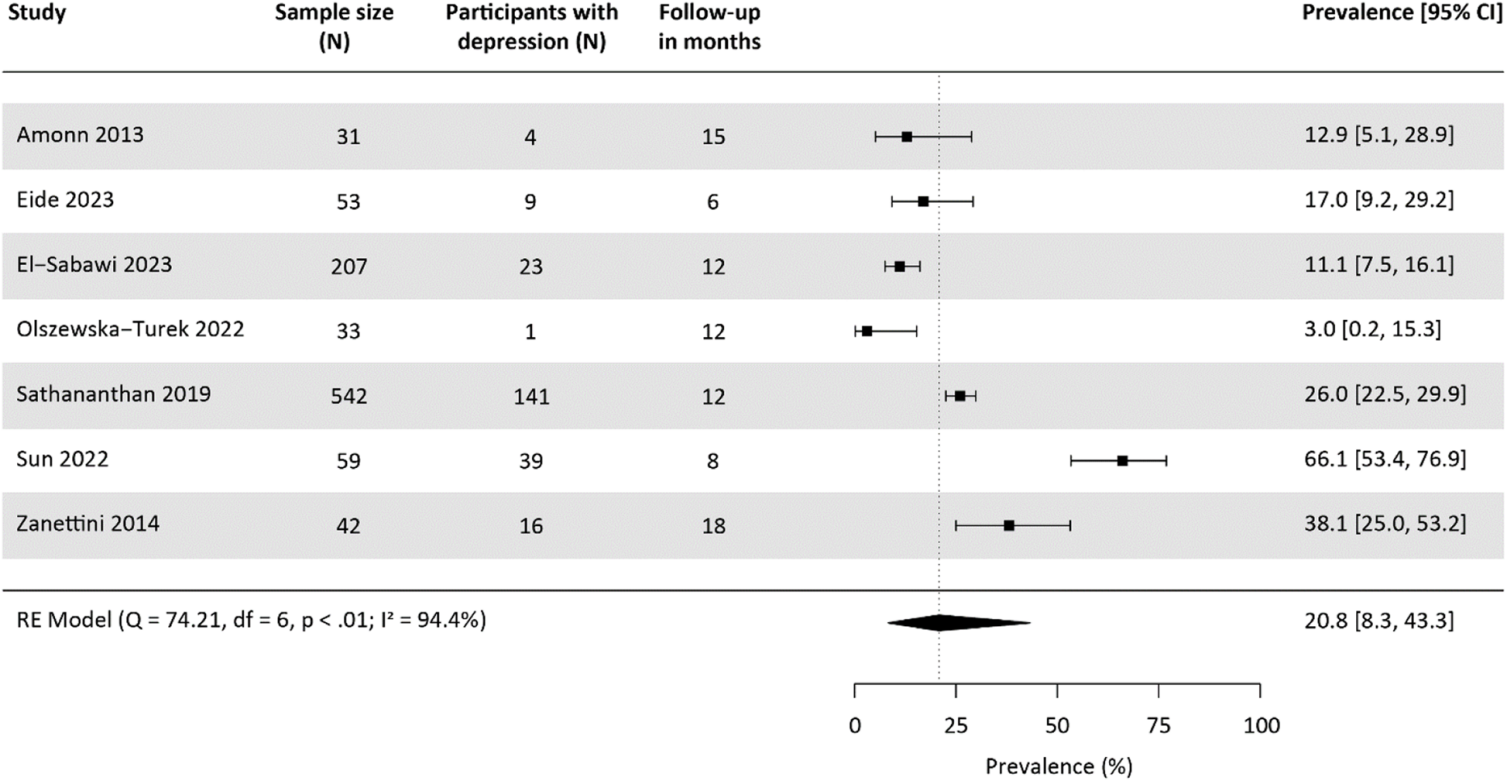
Meta-analysis on the prevalence of assessed depression at follow-up

Eight studies reported on follow-up prevalence of assessed depression with a follow-up duration ranging from six to 18 months [26, 34, 35, 50, 53, 60–62]. Due to overlapping samples, only seven studies were included in the meta-analysis, with the assessment instruments being the HADS (*n* = 3), a variant of the PHQ (*n* = 1) and a variant of the GDS (*n* = 3). The pooled follow-up prevalence of assessed depression based on a total of 977 study participants was 20.8% [95%-CI: 8.3, 43.3], with significant heterogeneity between studies (Fig. 4). Six of the included studies provided information on both baseline and follow-up prevalences. Of these, three observed a decrease and three an increase in prevalence of assessed depression, with overlapping CIs.

#### Comparison of prevalence estimates for diagnosed and assessed depression

Our analysis revealed a statistically significant difference between pooled prevalence rates of diagnosed and assessed depression, with the pooled prevalence of diagnosed depression being significantly lower than the pooled prevalence of assessed depression (*p* < 0.01) (details not shown).

### Course of depressive symptoms from pre- to post-TAVI

**Fig. 5:**
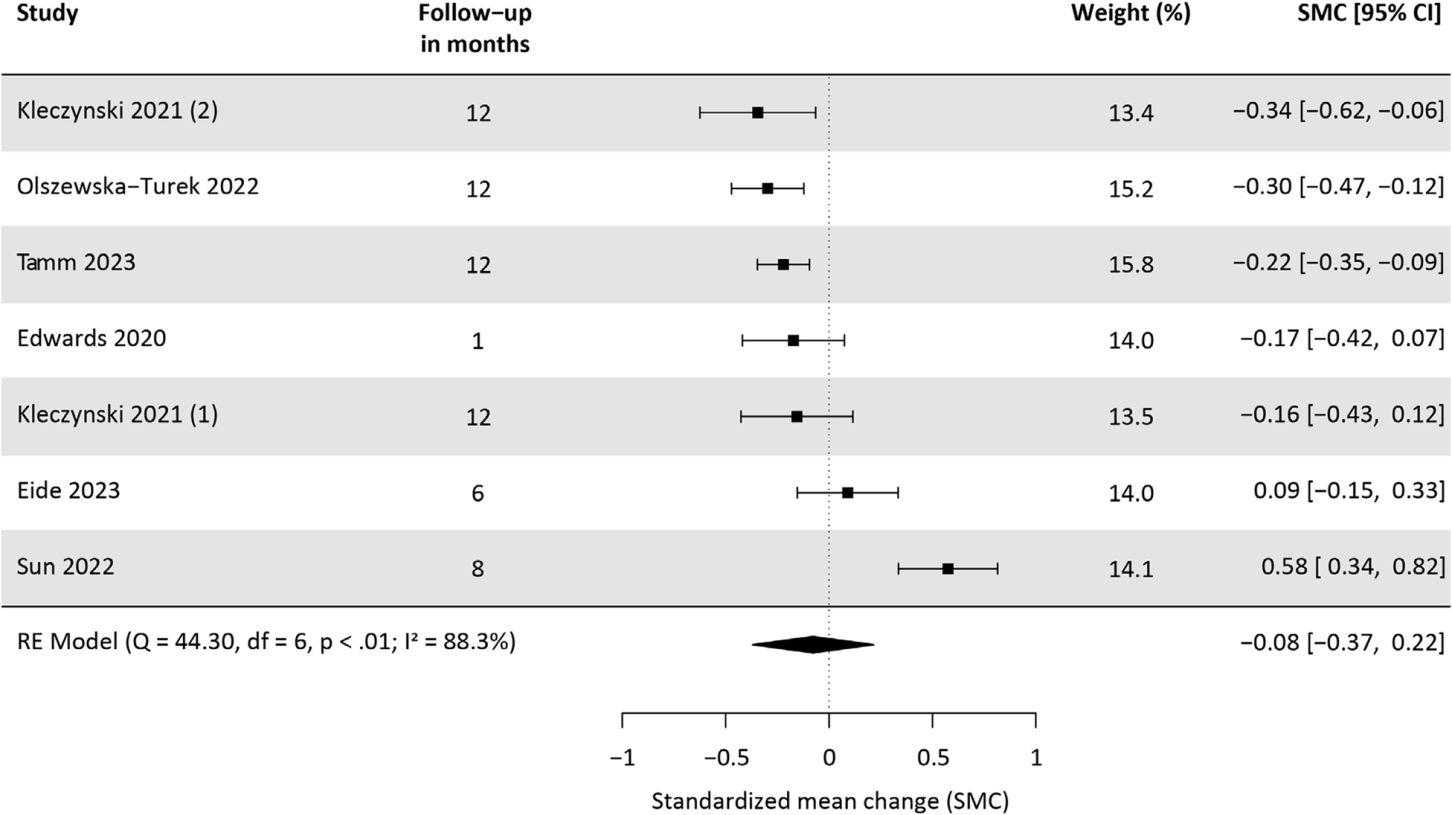
Meta-analysis on the standardized mean change of depressive symptoms from pre- to post-TAVI

Four studies reported mean and standard deviation [26, 32, 35, 61] and two studies median and interquartile range [40, 48] of depressive symptoms pre- and post-TAVI, with follow-up duration varying between one and 12 months. Kleczynksi 2021 [40] reported two estimates from independent treatment arms (treatment as usual (1) and cardiac rehabilitation (2)), and we included both in the meta-analysis. Depressive symptoms were assessed using the HADS (*n* = 3), a variant of the PHQ (*n* = 1), a variant of the GDS (*n* = 1) and a variant of the Beck Depression Inventory (BDI) (*n* = 1). The meta-analysis revealed a negative SMC from pre- to post-TAVI, which was not statistically significant. The Q-test and I^2^ statistic suggested substantial heterogeneity (Fig. 5), with a 95%-prediction interval of −0.85 to 0.70.

One study could not be included in the meta-analysis of SMC due to missing standard deviation of mean depression scale values from pre- to post-TAVI. This study reported a statistically significant decrease in depressive symptoms within 12 months post-TAVI [38]. Additionally, four studies were excluded because they collected baseline data at least 30 days post-TAVI. These studies observed a decrease in depressive symptoms from the time of admission to discharge in cardiac rehabilitation [39, 54, 55, 58], which was statistically significant in only two studies [54, 55].

### Sensitivity analyses

Generally, the sensitivity analyses described in the supplements supported the results of our main analyses and indicated robustness to alternative model specifications (See Supplementary Figures A1-A9, Additional File 1). Only the exclusion of Sun 2022 [35] as potential outlier from the meta-analysis of SMC produced divergent results, leading to a statistically significant negative pooled SMC of depressive symptoms, with no significant heterogeneity between studies (See Supplementary Figure A7, Additional File 1).

### Quality assessment

Studies that reported baseline prevalences of diagnosed depression met 78% of the quality criteria. The main issue identified in these studies was inappropriate recruitment of study participants due to self-selection (*n* = 4). In comparison, studies included in the meta-analysis of baseline and follow-up prevalences of assessed depression met 56% and 53% of the quality criteria, respectively. The main issues identified in these studies were self-selection of study participants (*n* = 15 and *n* = 8), inadequate sample size (*n* = 11 and *n* = 5), and inadequate response rate (*n* = 6 and *n* = 5). Studies included in the meta-analysis of SMC of depressive symptoms met 56% of the quality criteria. A common issue in those studies was self-selection of study participants (*n* = 7). Irrespective of outcome, coverage bias was unclear (*n* = 18) or present (*n* = 7) for almost all studies. Overall, we rate the study quality of studies included in this systematic review and meta-analysis as moderate, with studies reporting prevalence of diagnosed depression exhibiting high study quality (See Supplementary Material A4, Additional File 1).

### Certainty of evidence

We assigned a moderate confidence level to the prevalence estimates of both diagnosed and assessed depression. Despite observing large heterogeneity between studies, we rated the inconsistency as “not serious” because high heterogeneity between prevalence studies is inherent due to differing contexts and hence to be expected. We concluded that the true prevalence estimates are likely to be close to our pooled prevalence estimates.

The certainty of evidence regarding SMC in depressive symptoms from pre- to post-TAVI, on the other hand, is very low. We concluded serious risk of bias due to high dropout rates at follow-up in the included studies. Additionally, high heterogeneity among the studies led to inconsistent results, and estimates shifted notably in sensitivity analyses. The 95%-CIs varied widely, indicating potential for either a high decrease or a moderate increase in depressive symptoms, exhibiting serious imprecision.

## Discussion

To the best of our knowledge, this is the first systematic review and meta-analysis to determine the global prevalence and course of depression in older people with aortic valve stenosis undergoing TAVI. Applying rigorous, state-of-the-art statistical methods, we found a prevalence of 6.0% for diagnosed depression and of 18.5% for assessed depression, with a statistically significant difference between these prevalence estimates. Generally, these main results were robust against various alternative model specifications. Our results suggested a small, non-significant trend towards a decrease of depressive symptoms from pre- to post-TAVI, although the certainty of this finding is low.

Our prevalence estimates are consistent with previous studies examining the prevalence of depression in people with cardiovascular disease. A recent meta-analysis found a global prevalence of depression of 18.4% among people with cardiovascular disease, considering various definitions of depression, including clinical diagnoses and instrument-based assessments. This study also found a noticeably lower prevalence of diagnosed depression of 4.8% based on ICD codes, which supports our results [9]. Another recent meta-analysis focusing on prevalence of assessed depression in people with cardiovascular disease, however, observed a prevalence of 31.3% [8]. The discrepancies in these prevalence estimates could be explained by differences in regions, measurement instruments and disease entities. Firstly, the highest prevalence estimates for depression in people with cardiovascular disease were observed in countries from the Eastern Mediterranean Region or China, with prevalences of around 50% [63, 64]. However, our study mainly comprised studies from European countries and the USA. Secondly, meta-analyses that focused on assessed depression or on people with heart failure found higher pooled prevalences of depression. The World Health Organization reports a global prevalence of diagnosed depression for adults older than 60 years of 5.7% [18], which only slightly differs from our prevalence estimate of diagnosed depression. However, it is important to note that we included two studies that used treatment with antidepressants as sole indicator of a diagnosis of depression, potentially leading to an underestimation of the true prevalence of diagnosed depression. In summary, the pooled prevalence estimates in our analysis may appear comparatively low, but align with findings from studies that applied similarly defined diagnostic or assessment criteria in Western countries.

In general, the prevalence of diagnosed depression can be considered a more precise measure compared to assessed depression because of its established clinical relevance. Assessed depression, on the other hand, is more sensitive in identifying subclinical and undiagnosed depression cases, and in tracking changes in depressive symptoms. Therefore, the significant differences between the pooled prevalence estimates of diagnosed and assessed depression might indicate an under-diagnosis and, hence, an under-treatment, of depression among people undergoing TAVI. This is a concerning observation, as recent studies observed depression as an independent risk factor for increased mortality and lower quality of life after TAVI [33, 34, 44].

In our systematic review and meta-analysis of change in depressive symptoms, we observed a small trend towards a decrease in depressive symptoms from pre- to post-TAVI, although our meta-analysis did not yield a statistically significant result. This was unexpected, given that aortic valve stenosis is a slowly progressing disease, which is usually accompanied by severe symptoms that are likely to resolve post-treatment, potentially leading to an improvement in depressive symptoms. However, we identified the study of Sun 2022 [35] as potential outlier in all meta-analyses. Excluding this study from our meta-analysis of change in depressive symptoms revealed a statistically significant decrease in depressive symptoms after TAVI, with no between-study heterogeneity. It is worth noting that Sun 2022 [35] is the only study in our analysis conducted in China, while all other included studies were carried out in Western countries. In fact, compared with Western countries, Chinese people with aortic valve stenosis have greater calcification of leaflets and a higher frequency of bicuspid valve morphology [65]. Additionally, the uptake of TAVI has been slower compared to Western countries, with the first procedure only performed in 2010. That makes Chinese people undergoing TAVI a unique population [66]. Hence, we hypothesize that the discrepant results can be explained by these contextual differences between China and Western countries and thus, depressive symptoms may show improvements post-TAVI in people from Western populations. Nevertheless, the current state of research is still ambiguous and therefore, we argue that an improvement of depressive symptoms after treatment of aortic stenosis should not be taken for granted, underscoring the significance of comprehensive and high-quality follow-up care. Incorporating mental health interventions in cardiac rehabilitation may be an effective approach for improving depressive symptoms. [40, 54, 55]

Our findings point to a potentially high number of undetected depression cases among older people undergoing TAVI. This underscores the importance of addressing and recognising depression as a vital health concern in this patient group. While there is an increasing awareness of this issue within the scientific community, which is reflected by the emergence of psychocardiology as a research field, it has yet not been fully implemented in clinical guidelines. Specifically, disease management strategies for older people undergoing TAVI should be adapted to include regular screening and timely and adequate treatment of depression. The emphasis should be on guiding people with depression to specific disease management or cardiac rehabilitation programmes with a focus on mental health. Health care professionals involved in care of people undergoing TAVI should be made aware of the potentially high number of undiagnosed depression cases, and strive for coordinated cross-sectoral care for early detection and treatment. Future studies should focus on examining the effect of targeted depression treatment on both depressive symptoms and postoperative outcomes in RCTs to provide insights on effective interventions and their potential to enhance the overall well-being and prognosis of patients undergoing TAVI.

Our study has several notable strengths. These include the pre-registered study protocol, and the application of rigorous methods and comprehensive sensitivity analyses. In addition, separating diagnosed and assessed depression into two distinct independent meta-analyses, and including only studies that used validated instruments to assess depression, sets our work apart from other meta-analyses on the prevalence of depression.

Nonetheless, some limitations should be considered when interpreting our findings. Firstly, we observed a high degree of statistical heterogeneity in our meta-analyses. Differences in geographic regions, screening tools or diagnostic criteria may have contributed to this unexplained heterogeneity, but the number of studies was too small to perform subgroup analyses to explore this further. However, a high amount of between-study heterogeneity in meta-analyses of prevalence is rather common, because estimates are derived from observational studies within different settings [67]. Secondly, due to missing data, we had to calculate mean and standard deviation from median and interquartile ranges for some studies, which introduces a degree of uncertainty in our meta-analysis of SMC. However, this approach allowed us to include more studies, providing a more comprehensive evaluation of the course of depressive symptoms. Thirdly, we did not examine potential publication bias. For meta-analyses of prevalence, it was not deemed relevant, because the included studies were not primarily focused on reporting prevalence of depression, and prevalence data are typically not intended to test a specific effect or association. For the meta-analysis of change in depressive symptoms, the number of included studies was too small to reliably detect publication bias. However, studies reporting a significant increase or decrease in depressive symptoms might have a higher chance of being published, which could potentially bias the pooled estimate. Finally, due to a lack of research in some regions, the generalizability of our results is limited mainly to Western countries. Further studies are required to investigate prevalence and course of depression in non-Western countries. In addition, it is important to note that there may be underlying sex or gender differences in the prevalence and course of depression that we were unable to address, as none of the included studies explored sex or gender differences in the prevalence of depression. As this is an important research topic, we recommend that future studies investigate sex and gender differences in depression trajectories in older adults with cardiovascular diseases.

## Conclusions

In this systematic review and meta-analysis, we found a relevant prevalence of depression among older people with aortic valve stenosis before undergoing TAVI. A significant finding was that the pooled prevalence of diagnosed depression was considerably smaller than that of assessed depression, suggesting a substantial number of undetected depression cases. Additionally, we found that depressive symptoms did not change consistently from pre- to post-TAVI, although there was a small, non-significant trend towards a decrease in depressive symptoms. Our results underscore the need to promote awareness regarding depression in older people undergoing TAVI among healthcare professionals and highlight the importance of developing and implementing targeted interventions to address this issue effectively.

## Supplementary Information

Supplementary Material 1.

## Data Availability

The dataset generated and analysed during the current study is available in the Open Science Framework repository, [https://osf.io/97zy5/] (https://osf.io/97zy5).

## Abbreviations

BDI: Beck Depression Inventory
CI: Confidence interval
GDS: Geriatric Depression Scale
GLMM: Generalized linear mixed model
GRADE: Grading of Recommendations, Assessment, Development and Evaluation
HADS: Hospital Anxiety and Depression Scale
ICD: International Statistical Classification of Diseases and Related Health Problems
JBI: Joanna Briggs Institute
MINI: Mini-International Neuropsychiatric Interview
PCS: Prospective cohort study
PHQ: Patient Health Questionnaire
PRISMA: Preferred Reporting Items for Systematic Reviews and Meta-Analysis
PROSPERO: International prospective register of systematic reviews
RCS: Retrospective cohort study
RCT: Randomized controlled trial
RE: Random effects
SMC: Standardized mean change
TAVI: Transcatheter aortic valve implantation
UK: United Kingdom
USA: United States of America
