# Supplementary material for: Prevalence and course of depression in older people with aortic stenosis undergoing transcatheter aortic valve implantation – a systematic review and meta-analysis
Source: BMC Geriatr. 2025 Oct 6;25:756. doi: 10.1186/s12877-025-06402-w (PMC12502324; doi:10.1186/s12877-025-06402-w)
Supplement: Supplementary file 1 — Supplementary Material 1. [file 12877_2025_6402_MOESM1_ESM.docx]

**SUPPLEMENTAL ONLINE MATERIALS**

**Prevalence and course of depression in older people with aortic stenosis undergoing transcatheter aortic valve implantation – A systematic review and meta-analysis**

Verena Maschke^1^*, Ute Mons^1^, Valerie Lohner^1^

Affiliations:

^1^Cardiovascular Epidemiology of Aging, Department of Cardiology, Faculty of Medicine and University Hospital Cologne, University of Cologne, Germany.

*Corresponding author:

Verena Maschke

Cardiovascular Epidemiology of Aging, Department of Cardiology, Faculty of Medicine and University Hospital Cologne, University of Cologne, Germany

Kerpener Straße 62, 50937 Cologne, Germany

E-mail: verena.maschke@uk-koeln.de

Table of contents

[A1. PRISMA 2020 Checklist 3](#_Toc158883458)

[A2. Search strings 5](#_Toc158883459)

[A3. Sensitivity analyses 7](#_Toc158883460)

[A3.1. Methods 7](#_Toc158883461)

[A3.2. Results 8](#_Toc158883462)

[Figure A1 Meta-analyses on the prevalence of diagnosed depression at baseline with different transformation approaches. 8](#_Toc158883463)

[Figure A2 Meta-analyses on the prevalence of assessed depression at baseline with different transformation approaches. 8](#_Toc158883464)

[Figure A3 Meta-analysis on the SMC of depressive symptoms from pre- to post-TAVI with estimated means and SDs calculated with the quantile estimation method. 9](#_Toc158883465)

[Figure A4 Meta-analyses on the prevalence of diagnosed depression at baseline when separately excluding individual studies. 10](#_Toc158883466)

[Figure A5 Meta-analyses on the prevalence of assessed depression at baseline when separately excluding individual studies. 11](#_Toc158883467)

[Figure A6 Meta-analyses on the prevalence of assessed depression at follow-up when separately excluding individual studies. 12](#_Toc158883468)

[Figure A7 Meta-analyses on the SMC of depressive symptoms from pre- to post-TAVI when separately excluding individual studies. 13](#_Toc158883469)

[Figure A8 Meta-analyses on the SMC of depressive symptoms from pre- to post-TAVI with varying pre-post-correlations from 0% to 100%. 14](#_Toc158883470)

[Figure A9 Meta-analyses on the SMC of depressive symptoms from pre- to post-TAVI with varying pre-post-correlations from 0% to 100% after exclusion of a potential outlier. 14](#_Toc158883471)

[A4. Quality assessment 15](#_Toc158883472)

[Figure A10 Quality assessment of studies reporting prevalence of diagnosed depression at baseline. 15](#_Toc158883473)

[Figure A11 Quality assessment of studies reporting prevalence of assessed depression at baseline. 16](#_Toc158883474)

[Figure A12 Quality assessment of studies reporting prevalence of assessed depression at follow-up. 17](#_Toc158883475)

[Figure A13 Quality assessment of studies reporting SMC of depressive symptoms from pre- to post-TAVI. 17](#_Toc158883476)

[References 18](#_Toc158883477)

A1. PRISMA 2020 Checklist^1^

| **Section and Topic** | **Item #** | **Checklist item** | **Location where item is reported** |
| --- | --- | --- | --- |
| **TITLE** | | |  |
| Title | 1 | Identify the report as a systematic review. | Page 1 |
| **ABSTRACT** | | |  |
| Abstract | 2 | See the PRISMA 2020 for Abstracts checklist. | Page 1 |
| **INTRODUCTION** | | |  |
| Rationale | 3 | Describe the rationale for the review in the context of existing knowledge. | Page 2 |
| Objectives | 4 | Provide an explicit statement of the objective(s) or question(s) the review addresses. | Page 2 |
| **METHODS** | | |  |
| Eligibility criteria | 5 | Specify the inclusion and exclusion criteria for the review and how studies were grouped for the syntheses. | Page 3 |
| Information sources | 6 | Specify all databases, registers, websites, organisations, reference lists and other sources searched or consulted to identify studies. Specify the date when each source was last searched or consulted. | Page 3 |
| Search strategy | 7 | Present the full search strategies for all databases, registers and websites, including any filters and limits used. | Page 3 |
| Selection process | 8 | Specify the methods used to decide whether a study met the inclusion criteria of the review, including how many reviewers screened each record and each report retrieved, whether they worked independently, and if applicable, details of automation tools used in the process. | Page 3 |
| Data collection process | 9 | Specify the methods used to collect data from reports, including how many reviewers collected data from each report, whether they worked independently, any processes for obtaining or confirming data from study investigators, and if applicable, details of automation tools used in the process. | Page 4 |
| Data items | 10a | List and define all outcomes for which data were sought. Specify whether all results that were compatible with each outcome domain in each study were sought (e.g. for all measures, time points, analyses), and if not, the methods used to decide which results to collect. | Page 4 |
|  | 10b | List and define all other variables for which data were sought (e.g. participant and intervention characteristics, funding sources). Describe any assumptions made about any missing or unclear information. | Page 5 |
| Study risk of bias assessment | 11 | Specify the methods used to assess risk of bias in the included studies, including details of the tool(s) used, how many reviewers assessed each study and whether they worked independently, and if applicable, details of automation tools used in the process. | Page 5 |
| Effect measures | 12 | Specify for each outcome the effect measure(s) (e.g. risk ratio, mean difference) used in the synthesis or presentation of results. | Page 5 |
| Synthesis methods | 13a | Describe the processes used to decide which studies were eligible for each synthesis (e.g. tabulating the study intervention characteristics and comparing against the planned groups for each synthesis (item #5)). | Page 7 |
|  | 13b | Describe any methods required to prepare the data for presentation or synthesis, such as handling of missing summary statistics, or data conversions. | Page 5 |
|  | 13c | Describe any methods used to tabulate or visually display results of individual studies and syntheses. | Page 5 |
|  | 13d | Describe any methods used to synthesize results and provide a rationale for the choice(s). If meta-analysis was performed, describe the model(s), method(s) to identify the presence and extent of statistical heterogeneity, and software package(s) used. | Page 5 |
|  | 13e | Describe any methods used to explore possible causes of heterogeneity among study results (e.g. subgroup analysis, meta-regression). | Page 6 |
|  | 13f | Describe any sensitivity analyses conducted to assess robustness of the synthesized results. | Page 6 |
| Reporting bias assessment | 14 | Describe any methods used to assess risk of bias due to missing results in a synthesis (arising from reporting biases). | Page 4 |
| Certainty assessment | 15 | Describe any methods used to assess certainty (or confidence) in the body of evidence for an outcome. | Page 4 |
| **RESULTS** | | |  |
| Study selection | 16a | Describe the results of the search and selection process, from the number of records identified in the search to the number of studies included in the review, ideally using a flow diagram. | Page 7 |
|  | 16b | Cite studies that might appear to meet the inclusion criteria, but which were excluded, and explain why they were excluded. | Page 7 |
| Study characteristics | 17 | Cite each included study and present its characteristics. | Pages 7,8 |
| Risk of bias in studies | 18 | Present assessments of risk of bias for each included study. | Page 16 |
| Results of individual studies | 19 | For all outcomes, present, for each study: (a) summary statistics for each group (where appropriate) and (b) an effect estimate and its precision (e.g. confidence/credible interval), ideally using structured tables or plots. | Figures 1-3  Table 1 |
| Results of syntheses | 20a | For each synthesis, briefly summarise the characteristics and risk of bias among contributing studies. | Page 16 |
|  | 20b | Present results of all statistical syntheses conducted. If meta-analysis was done, present for each the summary estimate and its precision (e.g. confidence/credible interval) and measures of statistical heterogeneity. If comparing groups, describe the direction of the effect. | Pages 12-16 |
|  | 20c | Present results of all investigations of possible causes of heterogeneity among study results. | Page 21 |
|  | 20d | Present results of all sensitivity analyses conducted to assess the robustness of the synthesized results. | Page 16 |
| Reporting biases | 21 | Present assessments of risk of bias due to missing results (arising from reporting biases) for each synthesis assessed. | Page 16 |
| Certainty of evidence | 22 | Present assessments of certainty (or confidence) in the body of evidence for each outcome assessed. | Page 17 |
| **DISCUSSION** | | |  |
| Discussion | 23a | Provide a general interpretation of the results in the context of other evidence. | Page 18 |
|  | 23b | Discuss any limitations of the evidence included in the review. | Page 21 |
|  | 23c | Discuss any limitations of the review processes used. | Page 21 |
|  | 23d | Discuss implications of the results for practice, policy, and future research. | Page 22 |
| **OTHER INFORMATION** | | |  |
| Registration and protocol | 24a | Provide registration information for the review, including register name and registration number, or state that the review was not registered. | Page 3 |
|  | 24b | Indicate where the review protocol can be accessed, or state that a protocol was not prepared. | Page 3 |
|  | 24c | Describe and explain any amendments to information provided at registration or in the protocol. | Page 5 |
| Support | 25 | Describe sources of financial or non-financial support for the review, and the role of the funders or sponsors in the review. | Page 23 |
| Competing interests | 26 | Declare any competing interests of review authors. | Page 23 |
| Availability of data, code and other materials | 27 | Report which of the following are publicly available and where they can be found: template data collection forms; data extracted from included studies; data used for all analyses; analytic code; any other materials used in the review. | Page 23 |

# A2. Search strings

The search strings included variations of the terms “TAVI” and “depression”. We used the CoCoPop framework (Condition, Context, Population) to structure our research question, which has been established for systematic reviews of studies reporting prevalence data.^2^ We defined the condition of interest as depression or depressive symptoms before and/or after TAVI, diagnosed or assessed using validated instruments. We applied no restrictions regarding context and included all countries and settings. The target population consisted of people with AS receiving treatment with TAVI.

PubMed (MEDLINE)

| #1  #2  #3  #4  #5  #6  #7  #8  #9  #10  #11  #12  #13  #14  #15  #16  #17  #18  #19  #20  #21  #22  #23 | depress*  “mental health“  “mental disorder*“  MDD  “psychiatric disease*“  “psychiatric disorder*“  depression [MeSH Major Topic]  depressive disorder [MeSH Major Topic]  or/#1-#8  TAVI  TAVR  PAVI  PAVR  “transcatheter aortic valve implantation"  "transcatheter aortic valve replacement"  "percutaneous aortic valve implantation"  "percutaneous aortic valve replacement"  "aortic stenosis"  "aortic valve stenosis"  aortic valve stenosis [MeSH Major Topic]  transcatheter aortic valve replacement [MeSH Major Topic]  or/#10-#21  #9 and #22 |
| --- | --- |

Web of Science

| #1  #2  #3  #4  #5  #6  #7  #8  #9  #10  #11  #12  #13  #14  #15  #16  #17  #18  #19 | depress*  “mental health“  “mental disorder*“  MDD  “psychiatric disease*“  “psychiatric disorder*“  or/#1-#6  TAVI  TAVR  PAVI  PAVR  “transcatheter aortic valve implantation"  "transcatheter aortic valve replacement"  "percutaneous aortic valve implantation"  "percutaneous aortic valve replacement"  "aortic stenosis"  "aortic valve stenosis"  or/#8-#17  #7 and #18 |
| --- | --- |

APA PsycInfo

| S1  S2  S3  S4  S5  S6  S7  S8  S9  S10  S11  S12  S13  S14  S15  S16  S17  S18  S19  S20  S21  S22  S23 | depress*  “mental health“  “mental disorder*“  MDD  “psychiatric disease*“  “psychiatric disorder*“  DE "Depression (Emotion)"  DE "Major Depression"  DE "Mental Disorders"  DE "Mental Health"  or/S1-S10  TAVI  TAVR  PAVI  PAVR  “transcatheter aortic valve implantation"  "transcatheter aortic valve replacement"  "percutaneous aortic valve implantation"  "percutaneous aortic valve replacement"  "aortic stenosis"  "aortic valve stenosis"  or/S12-S21  S11 and S22 |
| --- | --- |

# A3. Sensitivity analyses

### A3.1. Methods

*Transformation approaches*

Firstly, recognising the ongoing debate about the most effective transformation approach in meta-analyses of proportions,^3,4^ we fitted two different random-effects (RE) models for sensitivity analysis: one with the Freeman-Turkey double-arcsine transformation and one with the single-arcsine transformation, both using restricted maximum likelihood (REML) estimation and the Knapp and Hartung method for confidence intervals (CIs).^5,6^

*Comparison of estimates of independent meta-analyses*

Secondly, considering the two approaches for comparing estimates of independent meta-analyses – a mixed-effects meta-regression model and a Wald-type test for comparing two separate meta-analyses^7^ – we performed the Wald-type test as sensitivity analysis. Furthermore, in our comparison of pooled prevalences of diagnosed and assessed depression, we included the prevalence estimates of assessed instead of diagnosed depression from studies reporting both diagnosed and assessed depression.

*Estimation of sample mean and standard deviation (SD)*

Thirdly, recognising the different approaches for estimating the sample mean and SD from median and its dispersion measures,^8^ we applied the quantile estimation method as sensitivity analysis.

*Pre-post-correlation and estimation approach*

Fourthly, we inserted different pre-post-correlations to check whether this would affect results. Additionally, we applied DerSimonian and Laird (DL) instead of REML method for meta-analysis of standardized mean change (SMC), as initially stated in our study protocol [details blinded for review].

*Outliers*

Lastly, across all meta-analyses, we excluded each study individually to check whether this would affect the summary estimates. We have placed a special focus on potential outliers. For the meta-analyses of prevalence, we classified studies as potential outliers if the CI of the prevalence estimate did not overlap with the CIs of any other included study. For the meta-analysis of SMC, we classified studies as potential outliers and/or influential points if the absolute externally studentized residuals were greater than 1.96 or if Cook’s distance was greater than one.^9^

### A3.2. Results

*Transformation approaches*

Different transformation methods revealed similar results for the pooled prevalence estimates of diagnosed and assessed depression. The prevalence estimates with CI and heterogeneity statistics for each transformation approach are shown in Figures A1 and A2.

### **Figure A1** Meta-analyses on the prevalence of diagnosed depression at baseline with different transformation approaches.


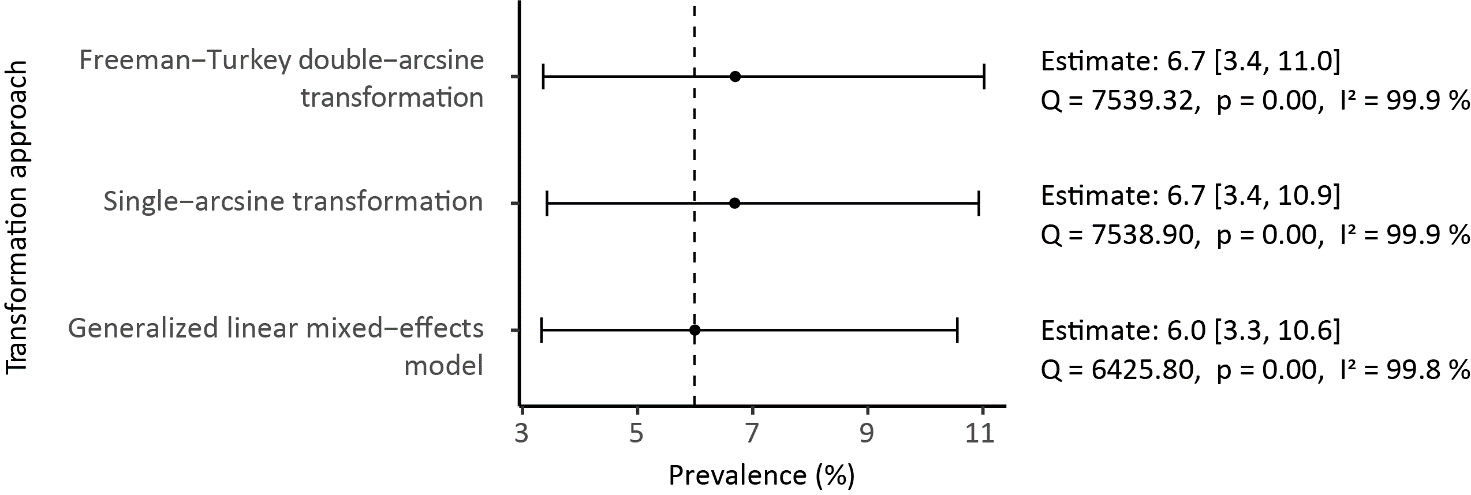


Note. The dashed vertical line marks the pooled prevalence estimate of our main analysis based on the generalized linear mixed-effects model.

### **Figure A2** Meta-analyses on the prevalence of assessed depression at baseline with different transformation approaches.


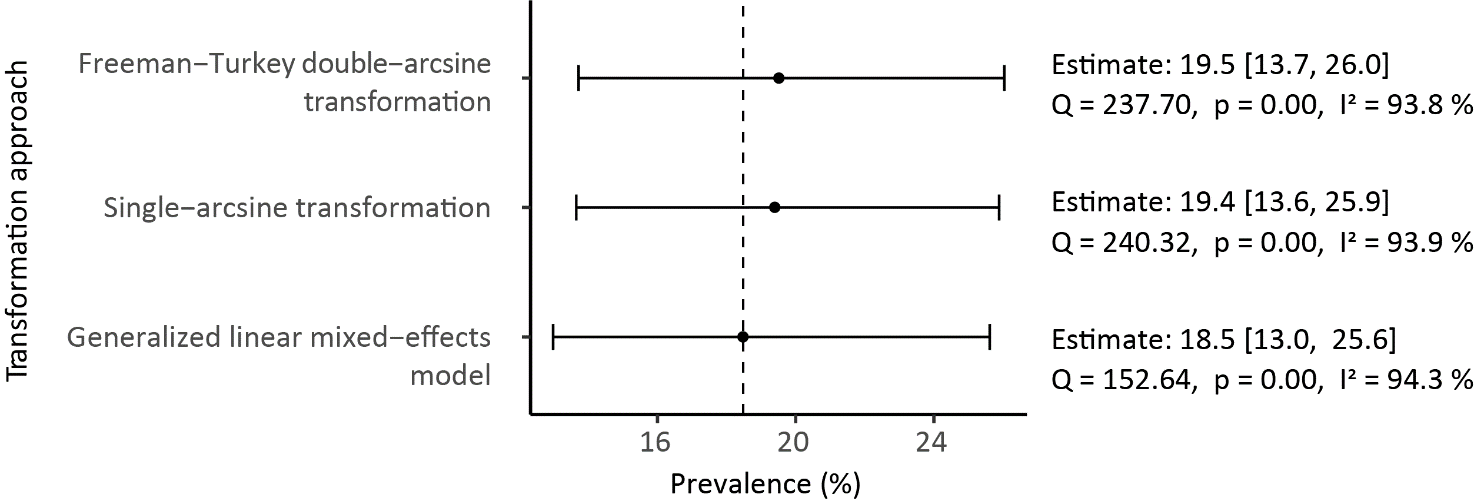


Note. The dashed vertical line marks the pooled prevalence estimate of our main analysis based on the generalized linear mixed-effects model.

*Comparison of estimates of independent meta-analyses*

The Wald-type test revealed significant differences between pooled prevalences of assessed and diagnosed depression (p < 0.01), supporting the results from our main analysis. We removed prevalence estimates of diagnosed instead of assessed depression to ensure independency of studies, which did not alter the results either (p< 0.01).

*Estimation of sample mean and SD*

When we derived estimated means and SD from the QE method, the pooled SMC did not significantly differ from zero and there was a significant amount of heterogeneity, supporting the results of our main analysis (see Figure A3).

### **Figure A3** Meta-analysis on the SMC of depressive symptoms from pre- to post-TAVI with estimated means and SDs calculated with the quantile estimation method.

*
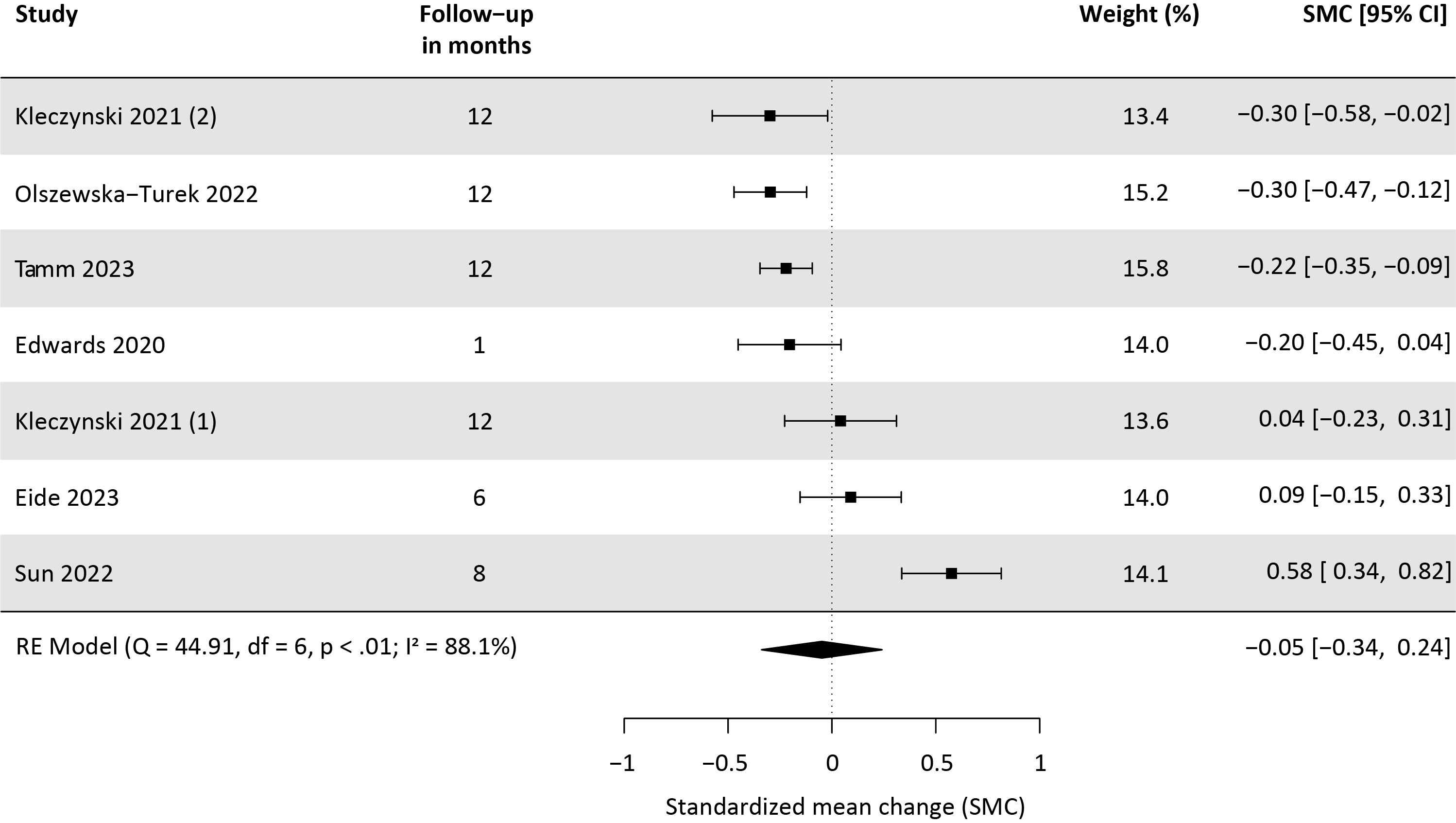
*

*Outliers*

For the meta-analyses of prevalence of diagnosed and assessed depression at baseline and follow-up (for assessed depression only), the step-by-step exclusion of individual studies did not yield considerably different results (see Figures A4-A6). As expected, we observed the strongest deviations when separately excluding the previously identified outliers. When simultaneously excluding those outliers, the prevalence estimates approach the results from our main analyses. However, when excluding Sun 2022^10^ from the meta-analysis of the SMC in depressive symptoms from pre- to post-TAVI, the resulting pooled SMC differed significantly from zero (p < 0.05) and heterogeneity reduced substantially to a non-significant amount (see Figure A7).

### **Figure A4** Meta-analyses on the prevalence of diagnosed depression at baseline when separately excluding individual studies.


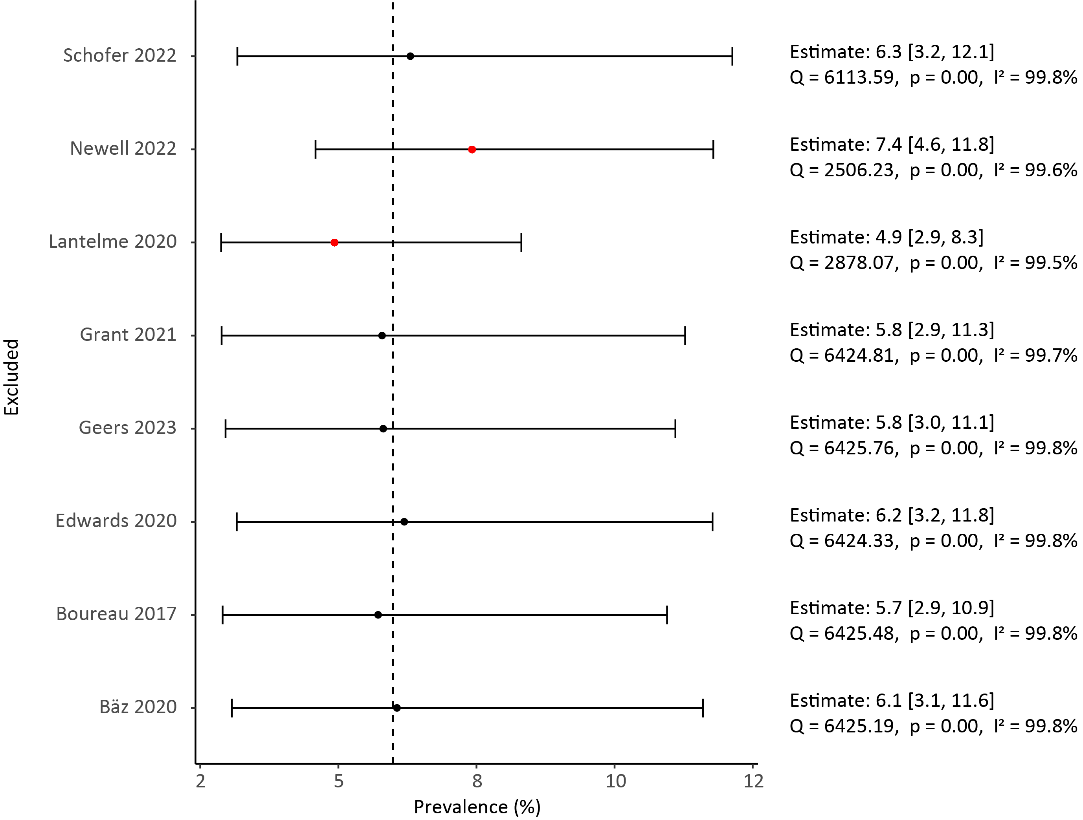


Note. Pooled prevalence estimates are coloured red, if we classified the excluded study as potential outlier. The dashed vertical line marks the pooled prevalence estimate of our main analysis (6.0%).

### **Figure A5** Meta-analyses on the prevalence of assessed depression at baseline when separately excluding individual studies.


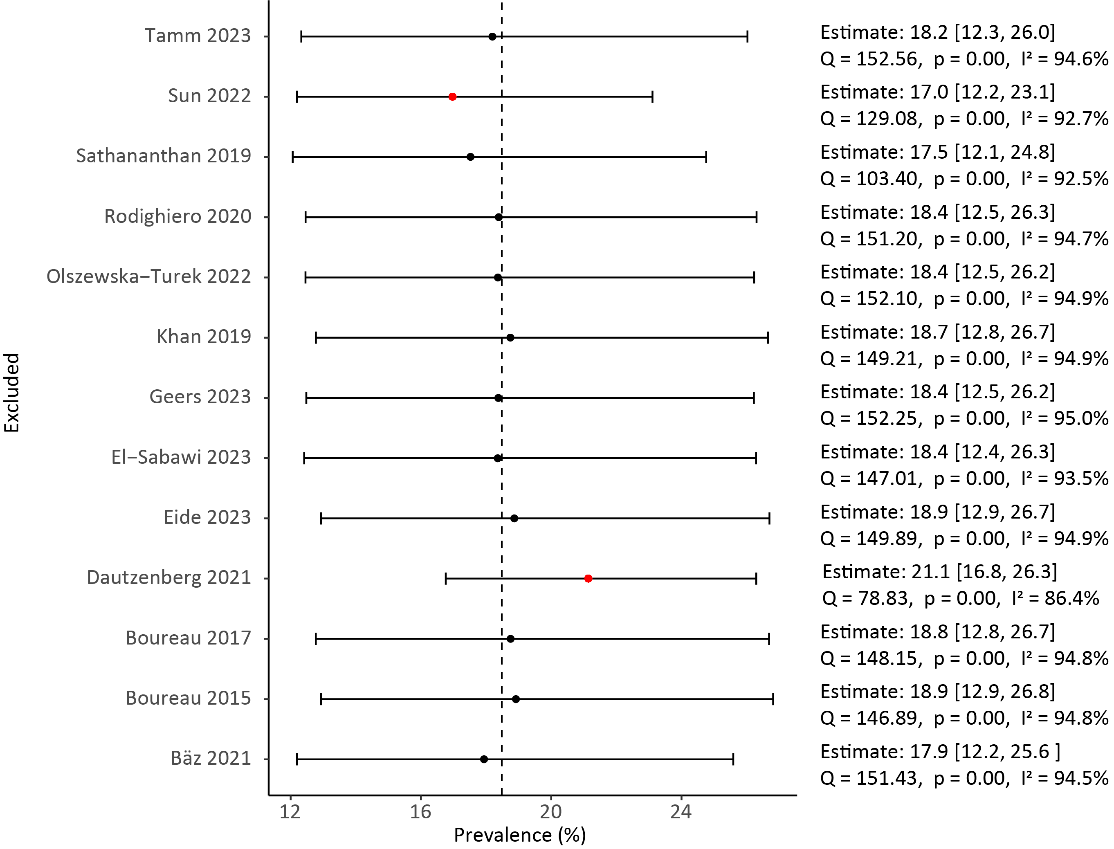


Note. Pooled prevalence estimates are coloured red, if we classified the excluded study as potential outlier. The dashed vertical line marks the pooled prevalence estimate of our main analysis (18.5%).

### **Figure A6** Meta-analyses on the prevalence of assessed depression at follow-up when separately excluding individual studies.


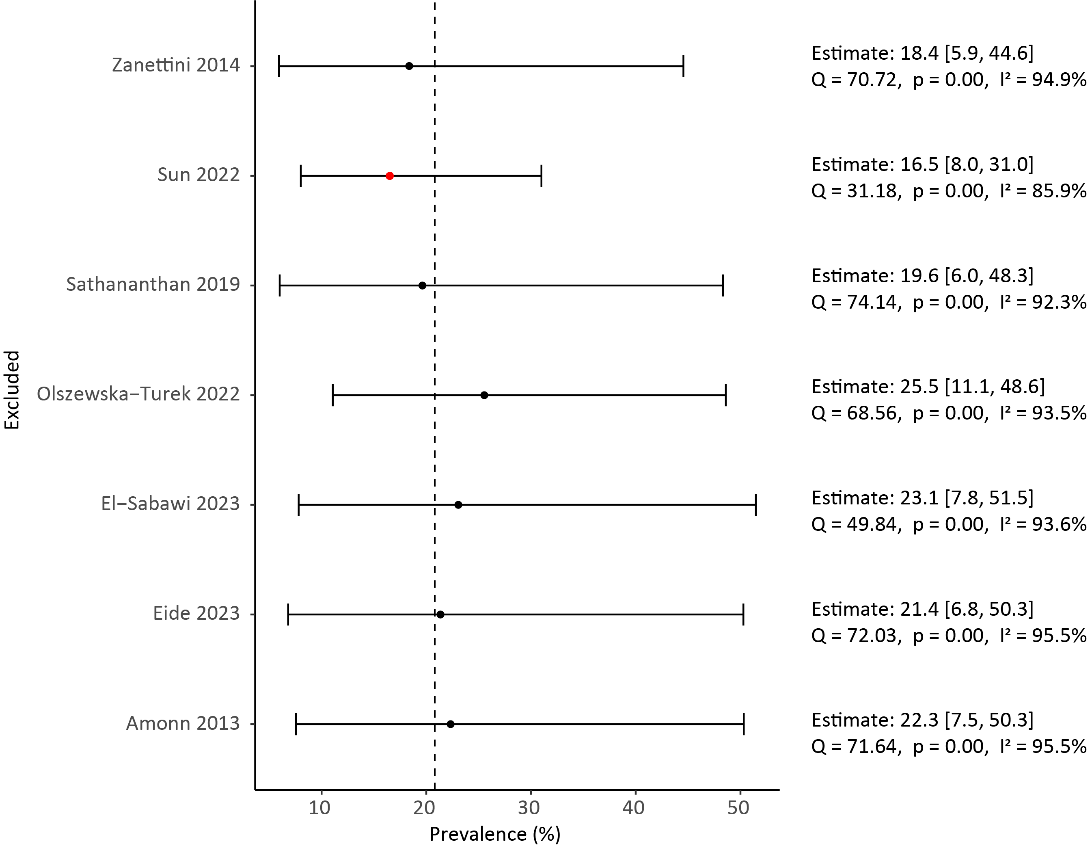


Note. Pooled prevalence estimates are coloured red, if we classified the excluded study as potential outlier. The dashed vertical line marks the pooled prevalence estimate of our main analysis (20.8%).

### **Figure A7** Meta-analyses on the SMC of depressive symptoms from pre- to post-TAVI when separately excluding individual studies.


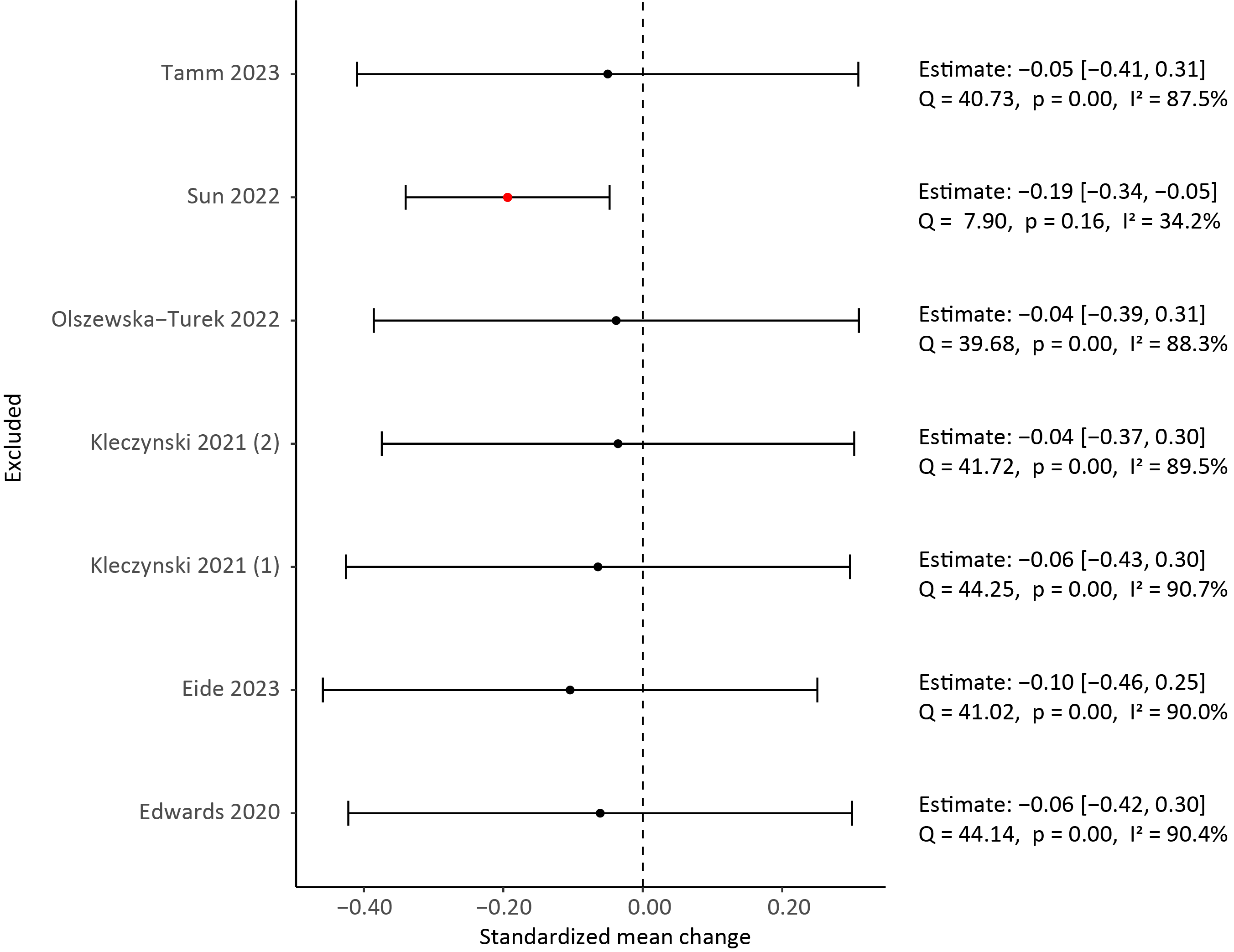


Note. Pooled estimates of the SMC are coloured red, if we classified the excluded study as potential outlier. The zero-effect line is shown as a dashed line.

*Pre-post-correlation and estimation approach*

With increasing pre-post-correlation, the pooled SMC and corresponding CIs hardly changed, up to a pre-post-correlation of about 95%, at which the effect sizes increased considerably and the CIs became very broad (see Figure A8). After excluding the potential outlier Sun 2022^10^, the same trend was observed, with the pooled SMC being statistically significant up to a pre-post-correlation of about 82% (see Figure A9). The use of DL instead of REML estimation method yielded the same SMC with corresponding CI.

### **Figure A8** Meta-analyses on the SMC of depressive symptoms from pre- to post-TAVI with varying pre-post-correlations from 0% to 100%.


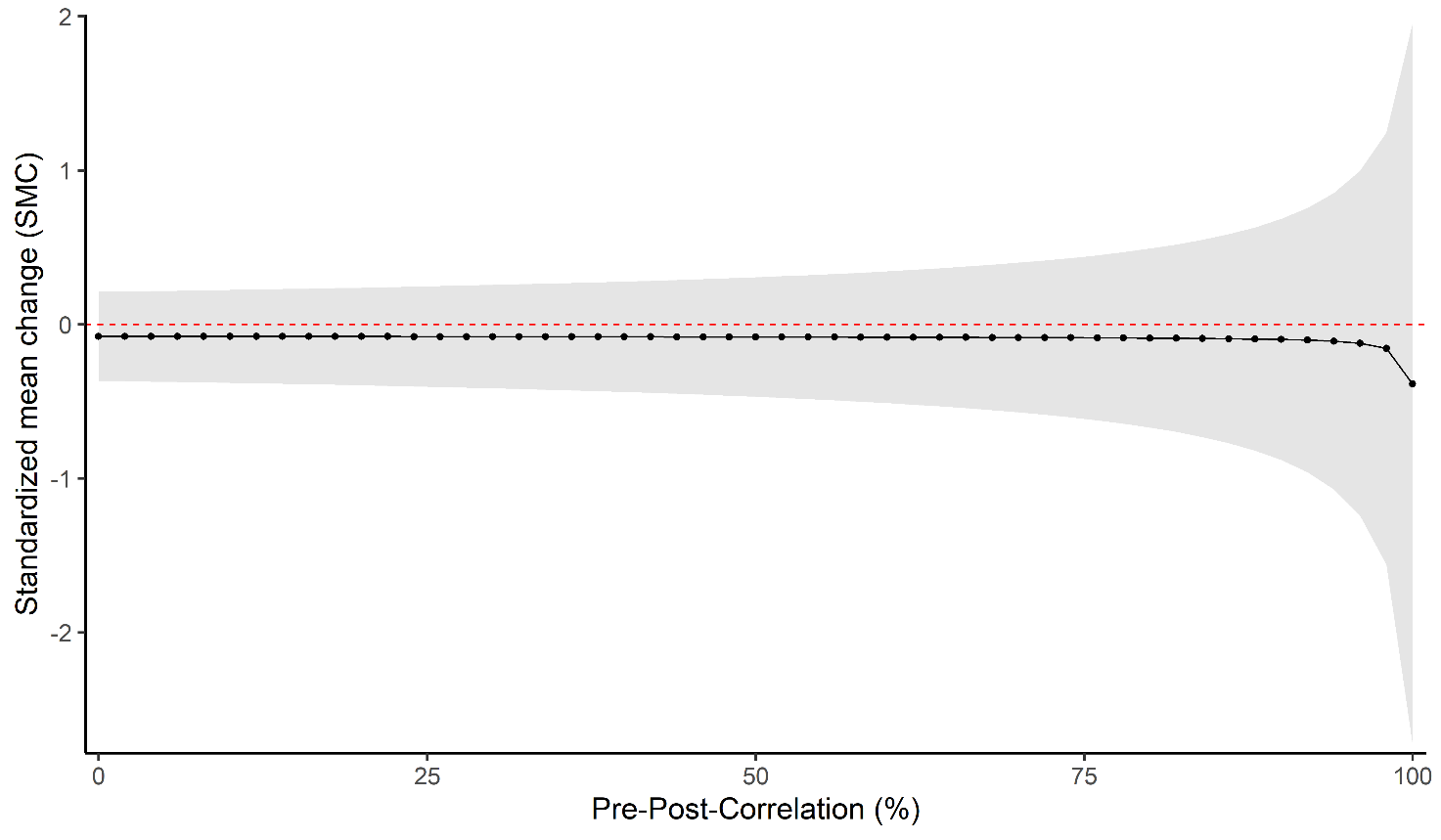


### **Figure A9** Meta-analyses on the SMC of depressive symptoms from pre- to post-TAVI with varying pre-post-correlations from 0% to 100% after exclusion of a potential outlier.


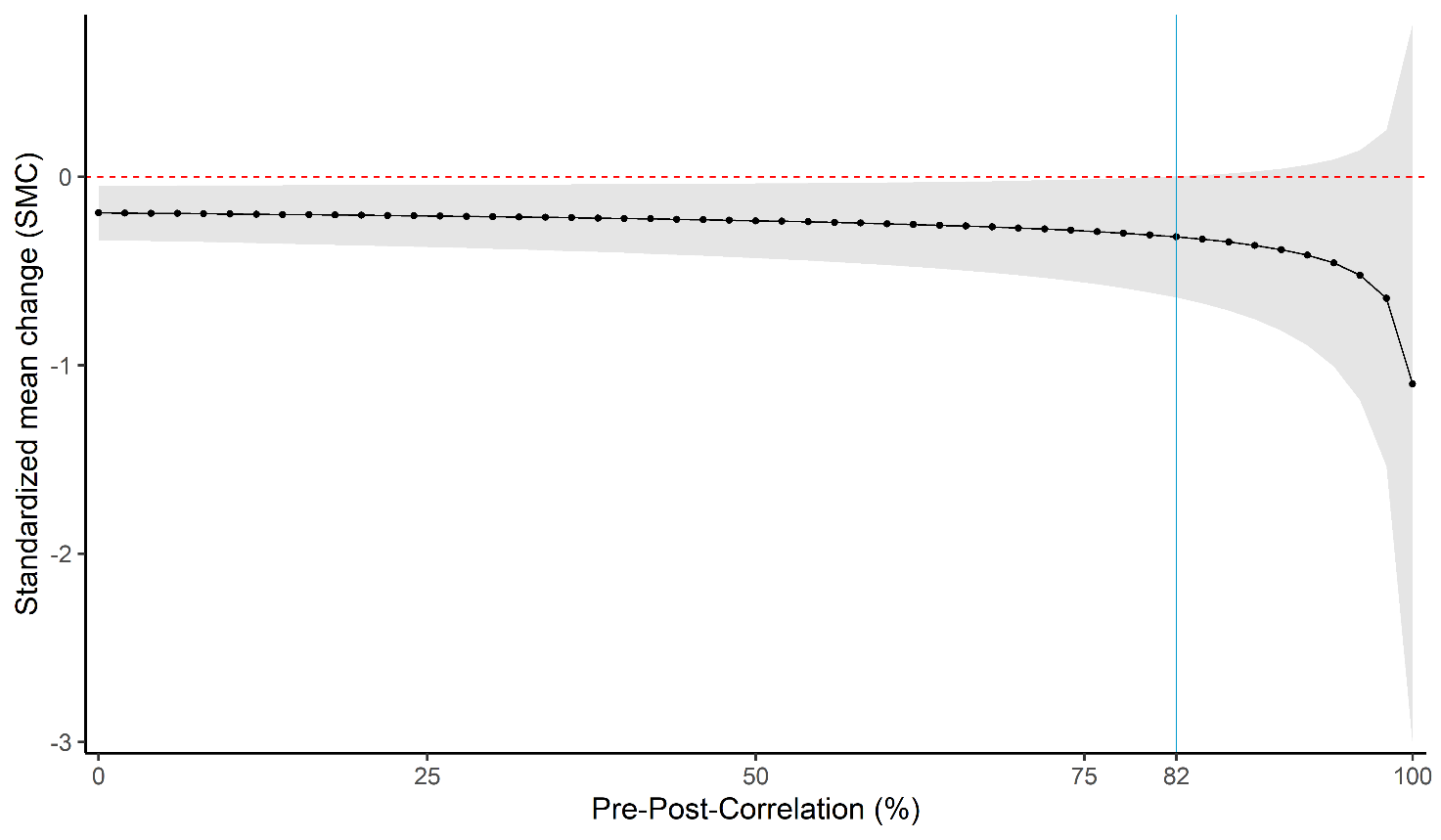
Note. The blue vertical line marks the pre-post-correlation above which the SMC is no longer statistically significant.

# A4. Quality assessment

The 2020 version of the JBI Critical Appraisal Checklist for prevalence studies addresses the following nine items: (1) appropriate sample frame to address the target population, (2) appropriate recruiting of study participants, (3) adequate sample size, (4) detailed description of study subjects and setting, (5) sufficient coverage of identified sample, (6) valid method for the identification of the condition, (7) standard and reliable way of measuring the condition, (8) appropriate statistical analysis, and (9) adequate response rate or management of low response rate.^11^ Those items were rated with “yes”, “no” or “unclear” for every study on an outcome level. There is currently no guideline on how to achieve an overall quality rating. We therefore assessed the overall quality of studies included in our main meta-analyses by calculating the average share of items answered with “yes”. The results are shown in Figures A10-A13 on an outcome level.

### **Figure A10** Quality assessment of studies reporting prevalence of diagnosed depression at baseline.

| **Study** | **1** | **2** | **3** | **4** | **5** | **6** | **7** | **8** | **9** | **Score (%)** |
| --- | --- | --- | --- | --- | --- | --- | --- | --- | --- | --- |
| Bäz 2020 | + | / | + | + | / | / | / | - | + | 44 |
| Boureau 2017 | + | / | + | + | + | + | + | - | + | 78 |
| Edwards 2020 | + | / | - | + | + | + | / | - | + | 56 |
| Geer 2023 | + | / | + | + | - | + | + | - | + | 67 |
| Grant 2021 | + | + | + | + | + | + | + | - | + | 89 |
| Krittanawong 2020 | + | + | + | + | + | + | + | - | + | 89 |
| Lantelme 2020 | + | + | + | + | + | + | + | - | + | 89 |
| Newell 2022 | + | + | + | + | + | + | + | - | + | 89 |
| Schofer 2022 | + | + | + | + | + | + | + | - | + | 89 |
| Shah 2019 | + | + | + | + | + | + | + | - | + | 89 |
| **Mean** |  |  |  |  |  |  |  |  |  | **78** |

Note. Green (+): „yes“; yellow (/): „unclear“; red (-): “no”. Score: the proportion of items rated with “yes”.

### **Figure A11** Quality assessment of studies reporting prevalence of assessed depression at baseline.

| **Study** | **1** | **2** | **3** | **4** | **5** | **6** | **7** | **8** | **9** | **Score (%)** |
| --- | --- | --- | --- | --- | --- | --- | --- | --- | --- | --- |
| Bäz 2021 | + | / | - | + | / | + | + | - | + | 56 |
| Bäz 2020 | + | / | - | + | / | + | + | - | + | 56 |
| Boureau 2015 | + | / | - | + | + | + | + | - | + | 67 |
| Boureau 2017 | + | / | - | + | + | + | + | - | + | 67 |
| Dautzenberg 2021 | + | / | + | + | + | + | + | - | + | 78 |
| Drudi 2018 | + | / | + | + | / | + | + | - | - | 56 |
| Eide 2023 | + | / | - | + | / | + | + | - | - | 44 |
| El-Sabawi 2023 | + | / | + | + | / | + | + | - | - | 56 |
| Geer 2023 | + | / | - | + | - | + | + | - | + | 56 |
| Khan 2019 | + | / | - | - | / | + | + | - | + | 44 |
| Olszewska-Turek 2022 | + | / | - | - | + | + | + | - | - | 44 |
| Rodighiero 2020 | + | / | - | + | / | + | + | - | + | 56 |
| Sathananthan 2019 | + | / | + | + | - | + | + | - | - | 56 |
| Sun 2022 | + | / | - | + | / | + | + | - | + | 56 |
| Tamm 2023 | + | / | - | + | / | + | + | - | - | 44 |
| **Mean** |  |  |  |  |  |  |  |  |  | **56** |

Note. Green (+): „yes“; yellow (/): „unclear“; red (-): “no”. Score: the proportion of items rated with “yes”.

### **Figure A12** Quality assessment of studies reporting prevalence of assessed depression at follow-up.

| **Study** | **1** | **2** | **3** | **4** | **5** | **6** | **7** | **8** | **9** | **Score (%)** |
| --- | --- | --- | --- | --- | --- | --- | --- | --- | --- | --- |
| Amonn 2013 | + | / | - | + | / | + | + | - | + | 56 |
| Drudi 2018 | + | / | + | + | / | + | + | - | - | 56 |
| Eide 2023 | + | / | - | + | / | + | + | - | - | 44 |
| El-Sabawi 2023 | + | / | + | + | / | + | + | - | - | 56 |
| Olszewska-Turek 2022 | + | / | - | - | + | + | + | - | - | 44 |
| Sathananthan 2019 | + | / | + | + | - | + | + | - | - | 56 |
| Sun 2022 | + | / | - | + | / | + | + | - | + | 56 |
| Zanettini 2014 | + | / | - | + | / | + | + | - | + | 56 |
| **Mean** |  |  |  |  |  |  |  |  |  | **53** |

Note. Green (+): „yes“; yellow (/): „unclear“; red (-): “no”. Score: the proportion of items rated with “yes”.

### **Figure A13** Quality assessment of studies reporting SMC of depressive symptoms from pre- to post-TAVI.

| **Study** | **1** | **2** | **3** | **4** | **5** | **6** | **7** | **8** | **9** | **Score (%)** |
| --- | --- | --- | --- | --- | --- | --- | --- | --- | --- | --- |
| Edwards 2020 | + | / | - | + | + | + | + | + | - | 67 |
| Eide 2023 | + | / | - | + | / | + | + | - | - | 44 |
| Kleczynski 2021 (DH) | + | / | - | + | / | + | + | + | + | 67 |
| Kleczynski 2021 (CR) | + | / | - | + | / | + | + | + | + | 67 |
| Olszewska-Turek 2022 | + | / | - | - | + | + | + | + | - | 44 |
| Sun 2022 | + | / | - | + | / | + | + | + | + | 56 |
| Tamm 2023 | + | / | - | + | / | + | + | + | - | 44 |
| **Mean** |  |  |  |  |  |  |  |  |  | **56** |

Note. Green (+): „yes“; yellow (/): „unclear“; red (-): “no”. Score: the proportion of items rated with “yes”. DH: discharged home. CR: cardiac rehabilitation.

# References

1. Page MJ, McKenzie JE, Bossuyt PM, Boutron I, Hoffmann TC, Mulrow CD*, et al.* The PRISMA 2020 statement: an updated guideline for reporting systematic reviews. *BMJ* 2021;**372**:n71. doi: 10.1136/bmj.n71

2. Munn Z, Stern C, Aromataris E, Lockwood C, Jordan Z. What kind of systematic review should I conduct? A proposed typology and guidance for systematic reviewers in the medical and health sciences. *BMC Med Res Methodol* 2018;**18**:5. doi: 10.1186/s12874-017-0468-4

3. Schwarzer G, Chemaitelly H, Abu-Raddad LJ, Rucker G. Seriously misleading results using inverse of Freeman-Tukey double arcsine transformation in meta-analysis of single proportions. *Res Synth Methods* 2019;**10**:476-483. doi: 10.1002/jrsm.1348

4. Doi SA, Xu C. The Freeman-Tukey double arcsine transformation for the meta-analysis of proportions: Recent criticisms were seriously misleading. *J Evid Based Med* 2021;**14**:259-261. doi: 10.1111/jebm.12445

5. Freeman MF, Tukey JW. Transformations Related to the Angular and the Square Root. *Annals of Mathematical Statistics* 1950;**21**:305-305. doi: 10.1214/aoms/1177729756

6. Anscombe FJ. The Transformation of Poisson, Binomial and Negative-Binomial Data. *Biometrika* 1948;**35**:246-254. doi: 10.1093/biomet/35.3-4.246

7. Rubio-Aparicio M, Sanchez-Meca J, Lopez-Lopez JA, Botella J, Marin-Martinez F. Analysis of categorical moderators in mixed-effects meta-analysis: Consequences of using pooled versus separate estimates of the residual between-studies variances. *Br J Math Stat Psychol* 2017;**70**:439-456. doi: 10.1111/bmsp.12092

8. McGrath S, Zhao X, Steele R, Thombs BD, Benedetti A, Collaboration DESD. Estimating the sample mean and standard deviation from commonly reported quantiles in meta-analysis. *Stat Methods Med Res* 2020;**29**:2520-2537. doi: 10.1177/0962280219889080

9. Viechtbauer W, Cheung MW. Outlier and influence diagnostics for meta-analysis. *Res Synth Methods* 2010;**1**:112-125. doi: 10.1002/jrsm.11

10. Sun J, Meng QT, Wang YW, Zhao WL, Sun FZ, Liu JH*, et al.* Comparison of the levels of depression and anxiety in elderly aortic stenosis patients treated with surgical or transcatheter aortic valve replacement. *J Cardiothorac Surg* 2022;**17**:141. doi: 10.1186/s13019-022-01888-6

11. Munn Z, Moola S, Riitano D, Lisy K. The development of a critical appraisal tool for use in systematic reviews addressing questions of prevalence. *Int J Health Policy Manag* 2014;**3**:123-128. doi: 10.15171/ijhpm.2014.71
